# A novel *TRPS1* mutation in a Moroccan family with Tricho-rhino-phalangeal syndrome type III: case report

**DOI:** 10.1186/s12881-017-0413-8

**Published:** 2017-05-03

**Authors:** W. Smaili, S. Chafai Elalaoui, S. Meier, M. Zerkaoui, A. Sefiani, K. Heinimann

**Affiliations:** 1 Centre de Génomique Humaine – Faculté de Médecine et de Pharmacie, Université Mohamed V, Rabat, Morocco; 2 Département de Génétique Médicale, Institut National d’Hygiène, 27 Avenue IbnBatouta, B. P.769, 11400 Rabat, Morocco; 3 Medical Genetics, University Hospital Basel, Schoenbeinstrasse 40, 4031 Basel, Switzerland

**Keywords:** Tricho-rhino-phalangeal syndrome type III, *TRPS1*, Exon 6, Novel missense mutation, Moroccan family

## Abstract

**Background:**

Tricho-rhino-phalangeal syndrome (TRPS) is an autosomal dominant disorder characterized by craniofacial and skeletal malformations including short stature, thin scalp hair, sparse lateral eyebrows, pear-shaped nose and cone shaped epiphyses. This condition is caused by haploinsufficiency of the *TRPS1* gene. Previous genotype-phenotype studies have correlated exon 6 missense mutations with TRPS type III, a severe form of type I with pronounced, facial characteristics, short stature and brachydactyly and differing from type II by the absence of exostoses and mental retardation.

**Case presentation:**

We report the first case of a Moroccan family, a father and his three children, in which the diagnosis of type III TRPS was suspected based on severe clinical and radiological features. Molecular analysis of the *TRPS1* gene revealed a novel missense mutation in exon 6, (p.Ala932Ser), located in the GATA-type DNA-binding zinc finger domain.

**Conclusion:**

Our observations in this kindred support the previous genotype-phenotype results suggesting that patients with more pronounced facial characteristics and more severe shortening of hands and feet are more likely to have mutation in exon 6 of *TRPS1*.

## Background

Tricho-rhino-phalangeal syndrome (TRPS) is a malformation syndrome characterized by short stature, sparse and slowly growing hair, a distinctive facies with rarefaction of lateral eyebrows, bulbous nasal tip, a high philtrum and thin upper lip. In addition, patients present cone-shaped epiphyses and severe generalized shortening of all phalanges, metacarpals and metatarsal bones [1]. This syndrome was first described by Giedion in 1966 [1]. Since then more than 120 patients have been described in the literature. Three subtypes of TRPS sharing all the common distinctive features have been described; type I which is the classical form of TRPS. Type II also called Langer-Giedion syndrome, which is distinguished from type I by the presence of multiple bony or cartilaginous exostoses in addition to mild mental retardation. This condition represents a contiguous gene syndrome on chromosome 8q24 [2]. Type III or Sugio-Kanjii Syndrome is characterized by severe shortness of all metacarpals, phalanges and stature without exostoses or mental retardation [3].

TRPS is usually inherited in an autosomal dominant manner [4], and caused by pathogenic mutations in the *TRPS1* gene (OMIM 604386), located on chromosome 8q23.3 and encoding a zinc finger transcriptional repressor with 2 potential nuclear localization signals and 9 different zinc-finger motifs [5]. The *TRPS1* protein appears to be involved in the regulation of the development of chondrocyte and perichondrium [6, 7]. Nonsense mutations are mostly found in patients with the TRPS I phenotype, while TRPS II is a contiguous gene deletion syndrome involving loss of the *TRPS1* and the EXT1 genes, the latter being mutated in multiple exostosis type I. Patients with the more severe phenotype, described as TRPS type III, have been found to carry missense mutations in the GATA-type DNA-binding zinc finger domain encompassing codons 903 to 953 [6]. Recently, genotype-phenotype studies showed that *TRPS1* exon 6 mutations, encompassing codons 902 to 941, may result in more pronounced facial characteristics and more severe shortening of hands and feet compared to mutations located in other *TRPS1* exons, but cohort numbers are still scarce to allow firm conclusions [8]. Several authors reported the management of TRPS short stature by growth hormone (GH) supplementation [9–11]. The results available to date are, however controversial.

Here, we report the first case of a Northern African family of 3 siblings and their father with a TRPS type III phenotype. The identification of a novel missense mutation, (p.Ala932Ser), in *TRPS1* exon 6 in this kindred provides further support that mutations in this exon may be correlated with a more pronounced features of the syndrome.

## Case presentation

**Fig. 1:**
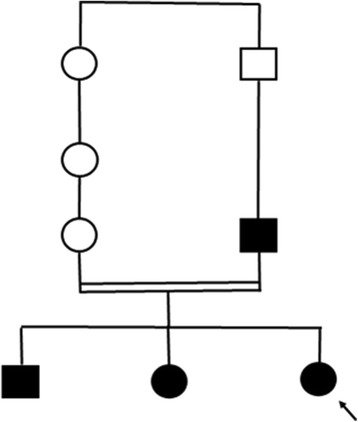
Pedigree of the family. The arrow indicates patient 1 which is the proband

We describe here a TRPS family with three affected siblings from a consanguineous marriage and their affected father (Fig. 1).

### Patient 1

**Fig. 2:**
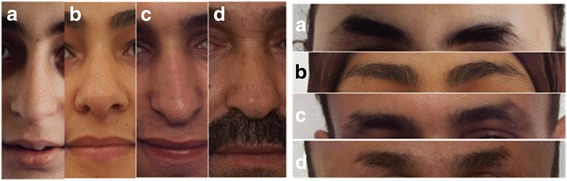
Typical dysmorphological features of TRPS III in the family members showing thick eyebrows with lateral rarefaction, characteristic pear-shaped nose, long philtrum and thin upper lip. **a**- Proband, **b**- Sister, **c**- Brother, **d**- Father

**Fig. 3:**
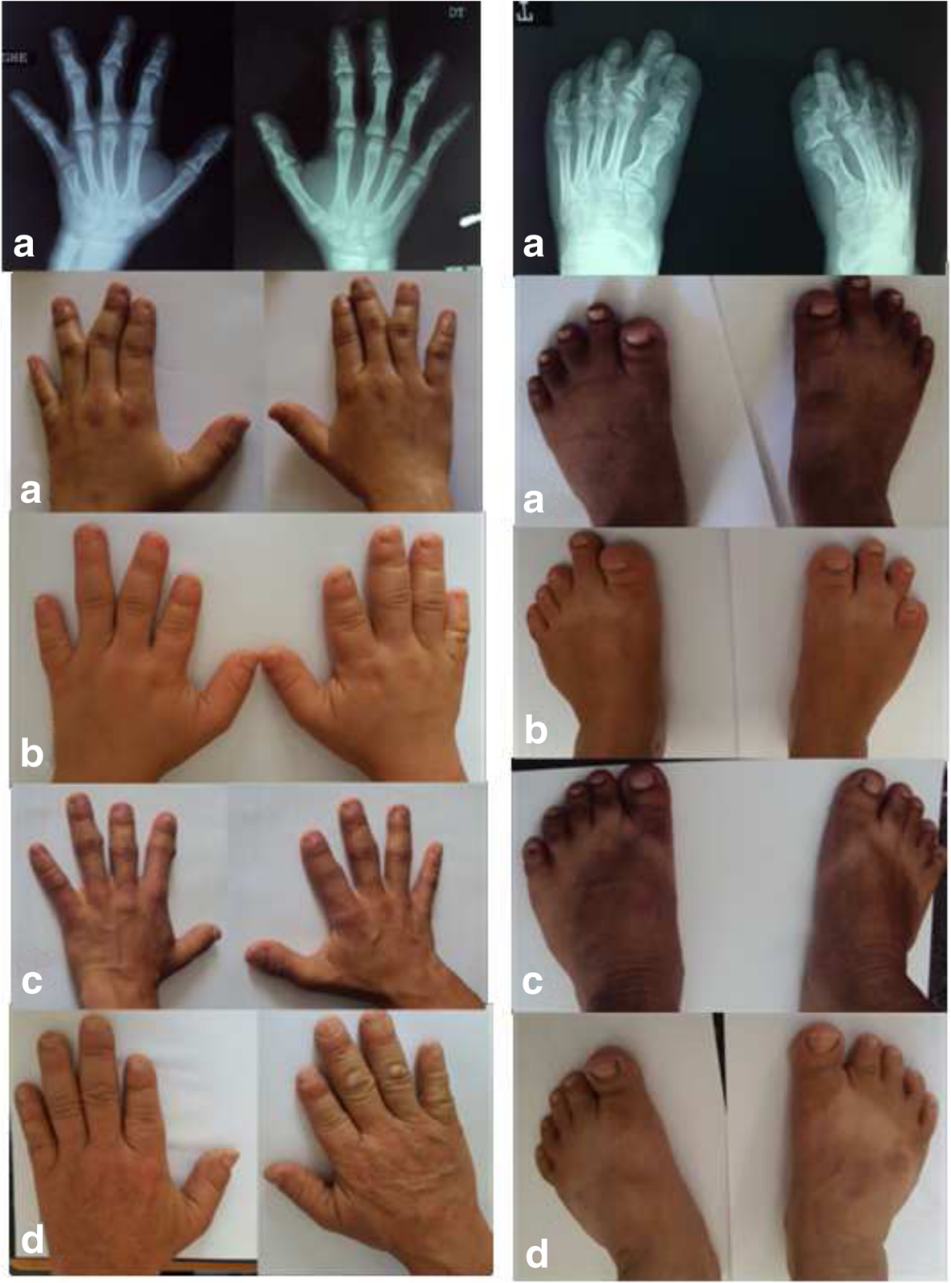
Characteristic hands and feet disclosing severe brachydactyly with syndactyly in the family members. **a**- Proband, **b**- Sister, **c**- Brother, **d**- Father. X-rays of the proband show misalignment of the middle and distal phalanges predominant at the third and fourth fingers with cone shaped epiphyses in hands and feet

A 13 years old girl was referred to the department of medical genetics in Rabat for short stature and brachydactyly. No prenatal investigations had been performed and the motor development was normal. Clinical examination found a proportionate short stature ≥ 3 SD (139 cm), sparse thin and hypo pigmented hair, thick eyebrows with lateral rarefaction, a characteristic pear-shaped nose, a long philtrum and a thin upper lip (Fig. 2a). No intellectual impairment was noticed. Extremities examination showed brachydactyly with bilateral axial deviation of third and fourth fingers, a short fifth metacarpal on the left, flat feet with partial syndactyly of the second and third toes on the right (Fig. 3a). Radiological examinations disclosed flattened femoral heads, poorly covered with a short and widened femoral neck predominantly on the right side in the pelvis radiograph. Hands’ radiographs showed misalignment of the middle and distal phalanges prevailing at the third and fourth fingers with irregular appearance of inverted V of the middle phalanges with cone-shaped corresponding epiphysis and a shortness of the fifth left metacarpal. Radiography of the feet found a bilateral misalignment of the distal phalanx of the hallux with a widened appearance of inverted V of the middle phalanges with cone shaped corresponding epiphysis. Spine and limbs radiographs in addition to the patient’s karyotype were all normal.

### Patient 2

The older sister of the proband was 18 years old; she presented a proportionate short stature (142 cm ≥ 3 SD) with thin, sparse and slow growing hair and the same distinctive facies characteristics as her sister, thick eyebrows and bulbous nasal tip (Fig. 2b). She showed also a brachydactyly with clinodactyly, deviation of the middle fingers on both hands, and partial syndactyly of the second and third toes on both feet (Fig. 3b). She had no intellectual impairment and no radiological exams were performed.

### Patient 3

The elder brother of the siblings was 21 years old; with no mental disorder, he presented a proportionate short stature (148 cm, ≥ 3 SD), thin scalp hair, rarefaction of lateral eyebrows and a typical pear-shaped nose with a long philtrum (Fig. 2c). He presented brachydactyly in both hands and feet with shortness of the right middle finger (Fig. 3c). The patient declined radiological exam of extremities.

### Patient 4

The father of patients 1, 2 and 3 presented with a severe proportionate short stature (141 cm, ≥ 3 SD), diffuse alopecia with fine hair, absence of lateral eyebrows, large beaked nose and a long philtrum with thin upper lip (Fig. 2d). He had short metacarpals with brachydactyly, axial deviation of the middle finger and racket nails (Fig. 3d). No radiological investigations were performed.

## Mutation analysis

Method: Following genetic counselling informed consent was obtained from all family members for both, molecular analysis and publication of clinical photographs. DNA was extracted from the three siblings and their two parents from EDTA blood samples using the QIAamp DNA Blood Mini Kit (Qiagen) according to the manufacturer’s protocol. Analysis of the entire coding region (exons 2 to 7, including intron/exon-boundaries) of the *TRPS1* gene (RefSeq: NM_014112.4) was performed by PCR amplification and bi-directional Sanger sequencing as previously described [12]. Gene dosage analysis was performed using the MLPA assay (P228, Lot No.: 0514, MRC Holland, NL). In silico prediction analysis was performed using SIFT (sift.jcvi.org) [13], MutationTaster (MT) [14] and PolyPhen-2 (PP2) [15] programs.

**Fig. 4:**
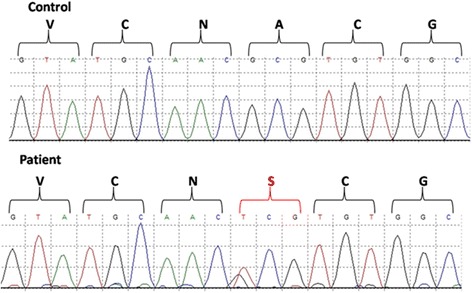
Electrophoregram showing above the wild-type sequence, and below the heterozygous sequence variant c.2794G > T identified in exon 6 of the *TRPS1* gene

In *TRPS1* exon 6 a heterozygous germline sequence variant, c.2794G > T, was identified in patient 1 (Fig. 4). Subsequent targeted mutation analysis of exon 6 of the remaining family members confirmed segregation of the sequence variant in the affected father (Case 4), and the two affected siblings (Cases 2 and 3). The healthy mother did not carry the sequence variant. This missense mutation leads to an amino acid change at codon 932, (p.Ala932Ser), replacing the nonpolar residue alanine by a polar one, serine. In silico prediction programs rat this mutation, occurring at an evolutionary highly conserved amino acid residue, as “disease causing” (MT), “damaging” (SIFT) and “probably damaging” (PP2, HumVar score: 0.999). Furthermore, two different missense mutations located at amino acid residue 932, (p.Ala932Thr) and (p.Ala932Val), have already been described as pathogenic in 4 index patients [6, 8, 16]. According to the ACMG criteria for classifying pathogenic variants [17], the (p.Ala932Ser) variant is classified as “likely pathogenic” (PM1, PM2, PM5, PP1, PP2, 149 PP3, PP4). This variant was absent in an in-house database of 770 exomes, and absent in the publicly available databases, including the Exome Aggregation Consortium [18], ExomeVariantServer [19] and dbSNP [20].

## Discussion

In 1966, Giedion reported several individuals with common manifestations including growth impairment, sparse hair, unusual face with characteristic shape of the nose and anomalies of the distal limbs, he named this entity tricho-rhino-phalangeal syndrome (TRPS) [1]. Three years after this description, Langer et al and Gorlin et al described patients with the same entity associated with multiple exostoses and developmental delay [21, 22]. Hall et al. suggested subdividing TRPS into two subtypes: TRPS I (OMIM 190350) for patients with normal development and without exostoses, and TRPS II or Langer-Giedion syndrome (OMIM 150230) for individuals with intellectual disorder and exostoses.

After this subdivision, several authors have proposed another subtype, TRPS III (OMIM 190351). This condition is associated with more marked growth delay and severe generalized shortening of all phalanges and metacarpals [3, 6, 23, 24]. Some authors discussed that TRPS III could be considered as part of TRPS I entity with more severe manifestation based on the small phenotypic differences and intrafamilial variability [6, 8, 23]. In our family, however, there was no evident intrafamilial clinical variability; growth delay ranged between ≥ 2.5 and ≥ 3 SD and severe brachydactyly in hands and feet was evident in all family members. Additional associated physical signs reported in TRPS type I, such as endocrine disturbance was not present in either of our family members [8, 25–27] In 2000, Momeni et al. reported *TRPS1* as the causal gene of TRPS [5]. This gene maps to 8q23.3 and encodes for a 141 kD multi zinc-finger transcription factor protein composed of 1294 amino acids with 9 putative zinc-finger motifs. The seventh zinc- finger motif binds to the GATA consensus cis element. *TRPS1* is assumed to be a nuclear regulator of chondrocyte proliferation and differentiation and hair follicles proliferation by homodimerization of the complex with GATA binding protein sequences in DNA to repress transcription of target genes [9, 28]. Thus *TRPS1* haploinsufficiency impairs endochondral cartilage differentiation and epithelial cell interactions in developing hair follicles by altering many signaling pathways as STAT3 and WNT/β catenin [7, 28–30]. Many studies reported mutations in the *TRPS1* gene and to date it appears to be the only gene associated with the TRPS phenotype [6, 8, 9, 27, 28]. According to the Human Genome Mutation Database, more than 120 pathogenic mutations correlated to TRPS phenotype have been reported, with most of them being nonsense or frameshift mutations. Genotype-phenotype studies showed that missense mutations associated to TRPS exclusively occurred in exons 6 and 7, with most of them being located in exon 6 which encodes a GATA-type DNA-binding zinc finger domain [8]. Furthermore, patients with missense mutations in exon 6 seem to have more pronounced facial characteristics and shortening of hands and feet compared to mutations located in other exons [6, 8]. Still, data on more TRPS families and patients are needed to allow firm conclusions. In our family a novel missense mutation in exon 6, (p.Ala932Ser), was clearly associated with a TRPS type III phenotype in all family members. The mutation is considered to be likely pathogenic since it involves an evolutionary highly conserved amino acid residue, is absent from controls (Exome Sequencing Project, 1000 Genomes Project), co-segregates with the phenotype and different missense changes at codon 932 have already been reported as pathogenic, however we could not deduce a clear overlap between the phenotype of our family and that of other patients carrying mutations in the same codon [6, 8]. The (p.Ala932Ser) mutation probably results in decreased affinity to DNA and thus may exert a dominant negative effect as a component of a multimeric protein complex [6, 16]. TRPS is a condition inherited in an autosomal dominant manner [4]. Approximately, half of mutations reported in the literature were inherited while the other half represents de novo mutations [31, 32]. In our family, it was evident that the affected father had transmitted the missense mutation to his 3 children in autosomal dominant manner. However some authors reported the occurrence of the disease in siblings from healthy parents suggesting an autosomal recessive transmission [33, 34]. Corsini et al. reported on a patient with a heterozygous mutation in the *TRPS1* gene whose healthy mothers was carrier of the mutation in the form of somatic mosaicism estimated at 10% in maternal blood-derived DNA using next generation sequencing. He proposed then a 5–10% risk of recurrence of *TRPS1* for healthy parents with an affected child, in all patients with presumed de novo mutations, based on the known recurrence risk in other dominant conditions with demonstrated germinal mosaicism, such as Cornelia de Lange syndrome and Rubinstein-Taybi syndrome [35].

## Conclusions

We report here the first case of a familial TRPS type III in Morocco with a novel *TRPS1* missense mutation, (p.Ala932Ser), associated with a more pronounced dysmorphic features and further supporting the phenotype-genotype studies suggesting an association between TRPS type III and missense mutations in *TRPS1* exon 6.

## Abbreviations

ACMG: Annual clinical genetics meeting
DNA: Deoxyribonucleic acid
EDTA: Ethylene-diamine-teraacetic acid
EXT: Exostosin
GH: Growth Hormone
IGF-1: Insulin-like growth factor 1
MLPA: Multiplex ligation-dependent probe amplification
MT: Mutation Taster
OMIM: Online Mendelian Inheritance in Man
PCR: Polymerase chain reaction
SD: Standard deviation
SIFT: Sorting intolerant from tolerant
STAT: Signal Transducer and Activator of Transcription
TRPS: Tricho-rhino-phalangeal syndrome.
WNT: Wingless integration site
